# Successful determination of imatinib re‐administration dosage by therapeutic drug monitoring in a case of chronic myeloid leukemia initiating dialysis for acute renal dysfunction

**DOI:** 10.1002/ccr3.4357

**Published:** 2021-08-16

**Authors:** Ryosuke Nakahara, Takahiro Sumimoto, Ryota Tanaka, Masao Ogata, Hiroki Itoh

**Affiliations:** ^1^ Department of Clinical Pharmacy Oita University Hospital Oita Japan; ^2^ Department of Hematology Oita University Hospital Oita Japan

**Keywords:** acute renal dysfunction, chronic myeloid leukemia, dialysis, Imatinib, therapeutic drug monitoring

## Abstract

Fixed dose regimen is currently the standard administration method for TKI. However, this case report indicated that TDM may by a useful approach to individualized dosing of TKI for the treatment of CML when initiating dialysis.

## Graphical abstract

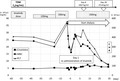

## 1 INTRODUCTION

Imatinib is used as first‐line treatment for chronic myeloid leukemia (CML) even in patients with impaired renal function. We successfully used therapeutic drug monitoring to determine the dose for re‐administration of imatinib in a CML patient who initiated dialysis for acute renal dysfunction associated with the initial imatinib therapy.

Imatinib is a first generation BCR‐ABL tyrosine kinase inhibitor (TKI) with anti‐leukemic activity against chronic myeloid leukemia (CML). In the International Randomized Study of Interferon and STI571 (IRIS), imatinib was superior to a combination of recombinant interferon alpha (IFNα) and low‐dose cytarabine with respect to the rates of cytogenetic and molecular responses^1^ as well as progression‐free survival (PFS) and overall survival.^2^ Given these clinical benefits, imatinib became the first‐line treatment for CML. Furthermore, randomized phase 3 trials have revealed that nilotinib and dasatinib, second‐generation BCR‐ABL TKIs, have superior efficacy compared with imatinib as first‐line treatment for chronic myelogenous leukemia in the chronic phase.^3^, ^4^ However, long‐term observation of 5 years revealed higher frequency of neovascular adverse effects associated with second‐generation TKIs than with imatinib.^5^, ^6^ From the adverse effect profile of the three TKIs, imatinib may be selected as first‐line treatment, taking into account patient background such as comorbid diseases. According to a Japanese package insert, typical adverse events of imatinib mesylate include nausea, vomiting, edema, tumor necrosis, muscle cramps, hematologic adverse effects, cardiovascular adverse effects, hepatic adverse effects, nephrotoxicity, and dermatologic adverse effects such as skin rash, pruritus, and petechiae. Initially, renal failure associated with imatinib was reported to be a rare event, occurring in less than 1.0% of the patients in the dose‐escalating studies of chronic phase and blast crisis CML.^7^, ^8^ Similarly, the Novartis Oncology Medical information website (www.oncologymedicalservices.com) reported renal function abnormalities in 1.6% of 1234 CML patients. However, there were no reports of renal failure among the 553 newly diagnosed CML patients treated with imatinib in the IRIS trial, with follow‐up up to 6 years. It is now clear that imatinib therapy is occasionally associated with potentially irreversible acute renal injury, and long‐term treatment may cause a clinically significant decrease in estimated glomerular filtration rate. In 105 patients receiving imatinib after prior interferon therapy, 7.0% developed acute kidney injury with mean decrease in glomerular filtration rate of 2.77 ml/min per 1.73 m^2^ per year, and 12% of patients developed chronic renal failure.^9^ In other cases, renal failure linked to imatinib is often reversible,^10^, ^11^ although hemodialysis is sometimes needed.^12^ Thrombotic thrombocytopenic purpura,^13^ acute tubular necrosis,^12^ tubular vacuolization,^14^ and partial Fanconi syndrome^15^ have been reported following imatinib therapy. At the onset of renal disorder or initiation of dialysis, treatment withdrawal or dose reduction of imatinib is required, but there are currently no guidelines for dose adjustment. This report describes a case in which the dose of re‐administration of imatinib was successfully determined by therapeutic drug monitoring (TDM) in a patient with CML who initiated dialysis for acute renal dysfunction associated with the initial imatinib therapy.

## 2 CASE PRESENTATION

**FIGURE 1:**
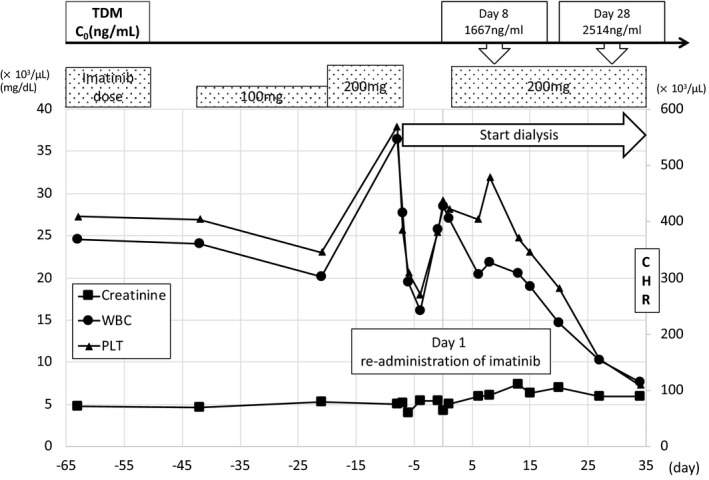
Clinical course. After onset of renal disorder and initiation of dialysis, TDM revealed that plasma imatinib level was within the therapeutic range. Thereafter, white blood cell count decreased to baseline level (3.3‐8.6 × 10^3^/μL), then reached complete hematologic response. TDM, therapeutic drug monitoring; C_0_, trough concentration; CHR, complete hematologic response; WBC, white blood cell count; PLT, platelet; X axis on the left represents WBC count (×10^3^/μL) and creatinine levels (mg/dL). Y axis on the right represents PLT count (×10^3^/μL)

An 88‐year‐old man treated with imatinib for CML was admitted to our hospital because of dyspnea and malaise. His current medical history was congestive heart failure, hypertension, type 2 diabetes, and chronic kidney disease G5A3. The patient underwent left nephrectomy for left renal cancer in November 2010. At discharge, decreased renal function was observed with blood urea nitrogen (BUN) 23 mg/dL and creatinine (Cr) 1.5 mg/dL. He was then regularly followed by his family doctor. In February 2014, a routine follow‐up blood test revealed white blood cell (WBC) count of 32.6 × 10^3^/μL, and he was referred to our hematology department in March 2014. At that time, renal function deterioration was recognized, with BUN 46 mg/dL and Cr 4.7 mg/dL, and he was clinically diagnosed with chronic phase Philadelphia‐positive CML, which was later confirmed by blood tests, bone marrow examination, and imaging findings. In April of the same year, imatinib was started at a dose of 100 mg. After 3 weeks, the dose was increased to 200 mg. Blood test during outpatient visits showed gradual deterioration of renal function, and he was scheduled to consult a nephrology department. In May of the same year, he complained of dyspnea and malaise and consulted his family doctor. However, he was unable to go the local hospital because of poor physical condition, and was transported to the emergency department of our hospital and was admitted. On the day of admission (day −8), BUN was 54 mg/dL and Cr was 5.1 mg/dL, indicating impaired renal function. At that time, ejection fraction (EF) was 49.6%, laboratory test values were 23.2 U/L for aspartate aminotransferase (AST), 28.8 U/L for alanine aminotransferase (ALT), and 15.1 U/L for γ‐glutamyl transpeptidase (γ‐GTP), SpO2 was 93.9% (room air). A plain chest radiograph suggested pulmonary edema. Therefore, he was admitted to the Department of Nephrology of our hospital for initiation of dialysis and treatment of respiratory failure. Imatinib was suspected to have caused the rapid decline in renal function, and administration was discontinued on the same day. He had dyspnea and was poorly oxygenated; SpO_2_ was 90% with oxygen administration at 10 L/min. Pleural effusion and pulmonary edema observed on chest radiograph were considered to be flooding due to exacerbation of chronic renal failure. A flexible double‐lumen (FDL) catheter was inserted through the right internal jugular vein and emergency dialysis was started. Blood pressure at admission was as high as 182/98 mm Hg, and nitroglycerin was administered at 2 mL/h and human atrial natriuretic peptide (hANP) at 0.75 mL/h. Respiratory distress gradually improved with administration of nitroglycerin and hANP, and water removal by dialysis. On day 2, 2 L/min of oxygen delivered via nasal cannula improved SpO_2_ to 98%, and administration of nitroglycerin and hANP was terminated. The patient was taking 5 mg of oral amlodipine at admission, and the dose was increased to 10 mg due to high blood pressure, and returned to 5 mg when blood pressure decreased by water removal. On day 1, SpO_2_ was maintained at 98% without oxygen administration, and oxygen therapy was terminated on the same day. Chronic changes in the kidney were observed based on the increase of Cr before admission and CT findings at admission, and maintenance dialysis was considered necessary in the future. Therefore, on day 1, a shunt was built in the left forearm, and maintenance dialysis became possible. After dialysis was initiated, a hematologist re‐administered oral imatinib on day 1. At that time, ejection fraction (EF) was 58.6%, laboratory test values were 26.3 U/L for aspartate aminotransferase (AST), 26.3 U/L for alanine aminotransferase (ALT), and 11.3 U/L for γ‐glutamyl transpeptidase (γ‐GTP), SpO2 was 97.0% (room air). A nephrologist consulted us regarding dosage regiment of imatinib and removal rate of imatinib by dialysis in patients with chronic kidney disease. Our search found no clear guidelines regarding the dose of imatinib for dialysis patients. Since imatinib is metabolized in the liver, and 67% is excreted in feces and 13% in urine, dosage reduction is not recommended even in patients with renal failure. However, several reports indicated that the same dose for patients with normal renal function is used in patients with renal failure, and that adverse effects were severe in dialysis patients due to high plasma imatinib concentration, necessitating dose reduction.^16^ Therefore, we suspected that the plasma concentration of imatinib was increased to some extent in this patient. We proposed to measure the plasma concentration of imatinib to monitor how dialysis affects the effects and side effects of imatinib. The dose used to start re‐administration was 200 mg/day, the same as that before treatment suspension. At that time (day 1), WBC was 27.1 × 10^3^/μL, PLT was 423 × 10^3^/μL, BUN was 40.7 mg/dL, and Cr was 5.0 mg/dL. Plasma concentration was determined by a high performance liquid chromatographic method as described previously.^17^ Using this method, the plasma concentration of imatinib was 1667 ng/mL (Figure 1) at the 8th day of re‐administration. This value was higher than the mean trough concentration of 1002 ng/mL reported to be effective in treating CML by Picard et al^18^ After confirming the TDM results, the attending physician decided to continue prescribing the same dose. After resuming imatinib, WBC counts declined markedly to 14.65 × 10^3^/μL on day 20. On day 28 (27 days after resuming administration), steady‐state plasma concentration was confirmed. The trough plasma concentration of imatinib was 2514 ng/mL (Figure 1). This value was lower than the mean trough concentration of 3180 ng/mL reported to be associated with a higher frequency of grade 3/4 adverse events such as neutropenia.^19^ Renal function did not exacerbate, and no other adverse events were observed. At that time, WBC was 10.24 × 10^3^/μL, PLT was 155 × 10^3^/μL, BUN was 48.0 mg/dL, and Cr was 6.0 mg/dL. After confirming the TDM results and no adverse events, the attending physician decided to continue prescribing the same dose. On day 35, complete hematologic response (CHR) was achieved with normalization of peripheral blood data and extramedullary lesions. CHR was achieved within 3 months after the start of treatment, corresponding to the criteria of optimal response to TKI treatment.^20^ On day 40, the patient's condition was stable with dialysis and imatinib therapy, and he was transferred to another hospital.

## 3 DISCUSSION

Imatinib is a first generation BCR‐ABL TKI, and has anti‐leukemic activity against CML. Treatment of CML has improved dramatically with the development of TKIs, including imatinib. However, the interindividual variability in adverse events and clinical efficacy as well as high drug cost remain major issues, and present a major obstacle to treatment. There is also an issue of reduced efficacy and safety with prolonged TKI administration. Therefore, TDM of TKIs is an important tool for CML treatment. We have previously reported a case report of successful determination of nilotinib dosage by TDM in a patient with CML developing hepatic dysfunction.^21^ The safety and efficacy of imatinib for CML have been reviewed.^22^ Maintaining an optimal trough plasma imatinib concentration is important for ensuring maximum efficacy in patients with CML.^22^ However, there are few case reports of effective dose adjustment of imatinib using TDM at the onset of adverse events. As one of the few case reports, Shibuya et al, when imatinib was administered at 400 mg/day to CML associated with maintenance dialysis patients, the C_max_ was 2,600 ± 800 ng/mL in normal subjects,^23^ but 4,950 ng/mL in dialysis patients was calculated.^16^ In addition, the imatinib removal rate by dialysis was as low as 6.3%, and it has been reported that plasma imatinib concentration increases gradually with continued administration in dialysis patients and may reach a peak of 5,800 ng/mL, which is considered to be the toxic level of imatinib.^16^ Furthermore, imatinib is metabolized in the liver by cytochrome P450 3A4 (CYP3A4). Since there are reports that uremic substances can reduce CYP3A4 activity in patients with chronic renal failure, caution should be exercised in long‐term use of drugs that are metabolized in the liver.^24^, ^25^ In this case, approximately one week after re‐administration imatinib when dialysis was initiated for renal impairment, TDM revealed that imatinib plasma level was within the therapeutic range (1667 ng/mL). TDM performed one month after imatinib was resumed with the patient on dialysis showed plasma imatinib concentration of 2514 ng/mL, which was within the safe range. These results also support previous report that the target trough concentration of imatinib is 1000‐3000 ng/mL.^22^ The large fluctuation in plasma imatinib concentration from day 8 to day 28 was thought to be due to the fact that a steady state was reached on day 28 after re‐administration. We believe that more frequent monitoring by TDM was necessary in this regard. After plasma imatinib concentration was confirmed by TDM, the physician decided to continue with the same dose. Treatment was continued without any adverse effects such as deterioration of renal function, and finally complete hematologic response was achieved 34 days after resuming imatinib treatment. Thus, TDM allowed maintenance of optimal plasma imatinib concentrations, which not only prevented the occurrence of adverse events, but also maintained clinical efficacy. As for the decrease in WBC and PLT levels after discontinuation of imatinib, we believe that the high WBC and PLT levels improved due to the resolution of the exacerbation of chronic renal failure and congestive heart failure caused by the initiation of dialysis with an FDL catheter during emergency hospitalization. This case report demonstrates successful determination of imatinib re‐administration dosage by TDM in a CML patient at initiation of dialysis for acute renal dysfunction. TDM may provide useful marker for individualized dosing of BCR‐ABL TKIs in the treatment of CML.

## CONFLICT OF INTEREST

There are no conflicts of interest to report.

## AUTHOR CONTRIBUTIONS

RN: analyzed data and wrote the manuscript. TS: analyzed data. MO: managed the patient. RT: wrote and reviewed the manuscript. HI: reviewed the manuscript. All authors read and approved the manuscript to be submitted.

## PATIENT CONSENT

Written informed consent was obtained from the patient for publication of this case report and accompanying clinical data.

## Data Availability

The data that support the findings of this study are available from the corresponding author, RN, upon reasonable request.
